# Inhibition of Angiotensin-Converting Enzyme Ameliorates Renal Fibrosis by Mitigating DPP-4 Level and Restoring Antifibrotic MicroRNAs

**DOI:** 10.3390/genes11020211

**Published:** 2020-02-18

**Authors:** Swayam Prakash Srivastava, Julie E. Goodwin, Keizo Kanasaki, Daisuke Koya

**Affiliations:** 1 Department of Diabetology & Endocrinology, Kanazawa Medical University, Uchinada, Ishikawa 920-0293, Japan; swayam.srivastava@yale.edu (S.P.S.); koya0516@kanazawa-med.ac.jp (D.K.); 2 Department of Pediatrics Yale University School of Medicine, New Haven, CT 06520, USA; julie.goodwin@yale.edu; 3 Division of Anticipatory Molecular Food Science and Technology, Kanazawa Medical University, Uchinada, Ishikawa 920-0293, Japan; 4 Shimane University Faculty of M2dicine, Internal Medicine 1, Enya-cho, Izumo, Shimane 693-8501, Japan

**Keywords:** diabetic nephropathy, ACE, ARBs, AcSDKP, DPP-4, EMT, EndMT, kidney fibrosis

## Abstract

Two class of drugs 1) angiotensin-converting enzyme inhibitors (ACEis) and 2) angiotensin II receptor blockers (ARBs) are well-known conventional drugs that can retard the progression of chronic nephropathies to end-stage renal disease. However, there is a lack of comparative studies on the effects of ACEi versus ARB on renal fibrosis. Here, we observed that ACEi ameliorated renal fibrosis by mitigating DPP-4 and TGFβ signaling, whereas, ARB did not show. Moreover, the combination of N-acetyl-seryl-aspartyl-lysyl-proline (AcSDKP), one of the substrates of ACE, with ACEi slightly enhanced the inhibitory effects of ACEi on DPP-4 and associated-TGFβ signaling. Further, the comprehensive miRome analysis in kidneys of ACEi+AcSDKP (combination) treatment revealed the emergence of miR-29s and miR-let-7s as key antifibrotic players. Treatment of cultured cells with ACEi alone or in combination with AcSDKP prevented the downregulated expression of miR-29s and miR-let-7s induced by TGFβ stimulation. Interestingly, ACEi also restored miR-29 and miR-let-7 family cross-talk in endothelial cells, an effect that is shared by AcSDKP suggesting that AcSDKP may be partially involved in the anti-mesenchymal action of ACEi. The results of the present study promise to advance our understanding of how ACEi regulates antifibrotic microRNAs crosstalk and DPP-4 associated-fibrogenic processes which is a critical event in the development of diabetic kidney disease.

## 1. Introduction

Diabetic nephropathy is the serious renal complication of diabetes and the leading cause of end-stage renal disease [1]. Kidney fibrosis is one of major outcome of diabetic nephropathy, caused by prolonged injury and dysregulation of the normal wound healing process with an excess deposition of extracellular matrix [2]. The origin of kidney fibroblasts still remains unclear and is a matter of ongoing debate [3]. Blood pressure control is essential to minimize the progression from diabetes to diabetic nephropathy [4,5]. Angiotensin-converting enzyme inhibitors (ACEis) and angiotensin II receptor blockers (ARBs) are two classes of anti-hypertensive agents that can effectively reduce the incidence of end-stage kidney disease and are first-line drugs for therapy in diabetic nephropathy [6,7,8]. However, there is a lack of comparative studies on the effects of ACEi versus ARB on renal fibrosis in diabetic nephropathy. Our research group reported that ACEi and ACEi in combination with N-acetyl-seryl-aspartyl-lysyl-proline (AcSDKP) prevent renal fibrosis by counteracting endothelial-to-mesenchymal transition (EndMT) in the kidneys of diabetic CD-1 mice whereas, ARB did not exert such protective effects in the kidneys of diabetic mice [9]. Understanding the mechanisms underlying the different response to ACEi and ARB treatment in mice with diabetic nephropathy is worth investigating. The dipeptidyl peptidase-4 (DPP-4) is found in its high level of expression and activity in the kidney [10]. Induced DPP-4 level in the kidneys is the key pro-fibrotic phenotype [11,12,13,14]. We found that induced level of DPP-4 interacts with integrin β1 [11,15]. Higher level of integrin β1 and DPP-4 interaction influences the TGF-β signaling and activates pro-endothelial-to-mesenchymal-transition (pro-EndMT) signaling [15]. 

Another study from our laboratory reveals that N-acetyl-seryl-aspartyl-lysyl-proline (AcSDKP) is the critical regulator of DPP-4 associated-TGF-β signaling-linked mesenchymal activation in the endothelial cells [9]. It is well-known that AcSDKP is produced by enzyme polyoligopeptidase (POP) on thymosin β4 protein (a constitutive protein) and is degraded by proteolytic processing of angiotensin converting enzyme (ACE) N-terminal [16]. ACEi treatment increases the plasma level of AcSDKP by fivefold [16]. Earlier researches in support of antifibrotic activity of AcSDKP includes the inhibition of TGF-β-induced expression of plasminogen activator inhibitor-1 and α2 collagen in human mesangial cells by inhibiting the smad 2/3 signaling pathway [17]; Shibuya et al. reported that AcSDKP prevented renal insufficiency and mesangial matrix expansion in diabetic db/db mice [18]; AcSDKP showed protective effects in various experimental animal models [19,20]. We here aimed to explore the ACEi and ARB actions on elevated DPP-4 level in regulating kidney fibrosis and to unravel the intermediary mechanisms involved. 

The actions and synthesis of microRNAs are tightly regulated; their expression level gets altered in the disease condition including in diabetes and diabetic nephropathy [21,22,23,24,25,26]. MiR-let-7 family members were involved in the antifibrotic mechanism of AcSDKP [9] whereas, miR-29 family members were implicated in the antifibrotic mechanism of linagliptin (DPP-4 inhibitor) [11]. AcSDKP acts by suppressing the higher DPP-4 level and restoring antifibrotic microRNA crosstalk between miR-29s and miR-let-7s [27]. ACEi alone treatment in the diabetic mice displayed higher expression level of miR-let-7 family whereas, ARB did not elevate [9]. Considering these, we conducted the experiments with ACEi and ARB and its effect on restoring antifibrotic microRNAs crosstalk and DPP-4-mediated active TGF-β signaling in the diabetic kidneys-associated with severe fibrosis. The present study, therefore, is a significant addition to the exiting knowledge of RAAS inhibitors and its implications on DPP-4 level and associated microRNAs regulations in the renal fibrosis.

## 2. Materials and Methods

### 2.1. Reagents and Antibodies

The AcSDKP was a gift from Dr. Omata (Asabio Bio Technology, Osaka, Japan). Imidapril (ACEi) and TA-606 (ARB) were provided (Mitsubishi Tanabe Pharma, Osaka, Japan) through an MTA. Linagliptin was provided by Boehringer Ingelheim (Ingelheim, Ingelheim am Rhein, Germany), with a MTA. The mouse monoclonal anti-human CD31 antibody (R&D Systems, Minneapolis, MN, USA) and the rat polyclonal anti-mouse CD31 antibody (EMFRET Analytics GmbH & Co. KG, Eibelstadt, Germany) were purchased. The polyclonal rabbit anti-αSMA antibody was obtained (Gene Tex, Irvine, CA, USA). The anti-TGF-β-receptor I, DPP-4 and p-smad3 antibodies were purchased (Sigma-Aldrich, St. Louis, MO, USA). Fluorescein-, rhodamine-, and Alexa 647-conjugated secondary antibodies were obtained (Jackson ImmunoResearch, West Grove, PA, USA). Anti-fibroblast specific proteins (FSP-1) and the HRP-conjugated secondary antibodies for Western blot detection were purchased (Cell Signaling Technology, Danvers, MA, USA). TGFβ2 were purchased (PeproTech, Rocky Hill, NJ, USA).

### 2.2. Animal Experiments

We utilized a fibrotic diabetic kidney disease model, i.e., streptozotocin (STZ)-treated CD-1 mice. Eight-week-old male CD-1 mice were obtained from Sankyo Lab Service (Tokyo, Japan). A single intraperitoneal injection of streptozotocin (STZ) (200 mg/kg) was given to the mice. We confirmed the induction of diabetes by a blood glucose level > 16 mM at 2 weeks after the STZ injection. Sixteen weeks after the induction of diabetes, the diabetic mice were divided into the following four groups: (ACEi-imidapril (2.5 mg/kg BW/day), AcSDKP (500 µg/kg BW/day using an osmotic mini-pump) + imidapril and TA-606 (ARB) (3 mg/kg BW/day), and vehicle treatment (using an osmotic mini-pump). Imidapril or TA-606 was provided in drinking water. In another set of experiments, we utilized two group of diabetic animal; the first group was treated as diabetic control (DM) while other was treated with linagliptin (5 mg/kg BW/day). All of the mice were euthanized by intraperitoneal injection of anesthesia ketamine/xylazine at 200/20 mg/kg/ body weight dose at 24 weeks after the induction of diabetes, and their blood pressure was monitored using the tail-cuff method with a BP-98A instrument (Softron Co. Beijing, China) within a week before euthanasia. The blood of each animal was withdrawn from the retro-orbital plexus and kidney tissues were collected. 

The experiments in the methods sections were carried out at Kanazawa Medical University in accordance with approved university animal protocols (protocol number 2014-89; 2013-114 and 2014-101). Authors confirm that all the experiments were performed in accordance with Japanese guidelines and regulations for scientific and ethical experimentation.

### 2.3. Immunofluorescence

Frozen kidney sections (5 μm) were used for immunofluorescence; double positive labeling with CD31/DPP-4 and DPP-4/αSMA was measured. Briefly, frozen sections were dried and placed in acetone for 10 min at −30 °C. Once the sections were dried, they were washed twice in phosphate-buffered saline (PBS) for 5 min and then blocked in 2% bovine serum albumin/PBS for 30 min at room temperature. Thereafter, the sections were incubated in primary antibody (CD31/DPP-4 (1:100) and DPP-4/αSMA (1:100) for 1 h and washed in PBS (5 min) three times. Next, the sections were incubated with the secondary antibodies for 30 min, washed with PBS three times (5 min each), and mounted with mounting medium with DAPI (Vector Laboratories, Burlingame, CA). The immune-labeled sections were analyzed by fluorescence microscopy (Axio Vert.A1, Carl Zeiss Microscopy GmbH, Jena, Germany). For each mouse, original magnification of 400× pictures were obtained from six different areas, and quantification was performed.

### 2.4. In Vitro Experiment in Endothelial Cells

Human dermal microvascular endothelial cells (HMVECs, Lonza, Basel, Switzerland) cultured in EGM medium were used in this experiment. When the HMVECs on the adhesion reagent (Kurabo medical, Osaka, Japan) reached 70% confluence, 5 ng/ml recombinant human TGFβ2 for 48 h was placed in the experimental medium (HuMedia-MVG in serum-free RPMI at a 1:3 ratio) with or without ACEi-(100 nM) and AcSDKP-(100 nM)+ACEi-(100 nM). AcSDKP-(100 nM) and ACEi-(100 nM) were pre-incubated 2h before TGFβ2 stimulation. In the control well, vehicle (DMSO) was added (3 × 10^−5^ dilution of DMSO in final concentration). In another set of experiments, HMVECs were treated with TGFβ2 in the experimental medium with or without ARB-(100 nM). ARB-(100 nM) were pre-incubated 2 h before TGF-β2 stimulation. The protein lysate was harvested for western blot analysis. 

Smad3 phosphorylation was analyzed in HMVECs treated with 30 with above inhibitors. For the gene expression analysis, HMVECs were treated with TGFβ2 in the experimental medium with or without ACEi-(100 nM), AcSDKP-(100 nM)+ACEi-(100 nM) and ARB-(100nM). AcSDKP-(100 nM), ACEi-(100 nM) and ARB-(100 nM) were pre-incubated 2 h before TGFβ2 stimulation. In another experiment, HMVECs were treated with TGFβ2 in the experimental medium with or without POPi (S17092-5 µM). mRNA was extracted using Qiagen mirneasy mini kit.

### 2.5. Western Blotting

Protein lysates were denatured in a SDS sample buffer at 100 °C for 5 min. After centrifugation (17,000× g for 10 min at 4 °C), supernatants were separated on SDS-polyacrylamide gels and blotted onto PVDF membranes (Pall Corporation, Pensacola, FL, USA). The semidry method was used to transfer the proteins and they were probed with the indicated antibodies: anti-TGF-β-receptor I (Sigma-Aldrich (St. Louis, MO, USA), 1:1000), anti-DPP-4 (Sigma-Aldrich (St. Louis, MO, USA), 1:1000), p-smad3 (Sigma-Aldrich (St. Louis, MO, USA), 1:500), smad3 (Cell Signaling Technology (Danvers, MA, USA), 1:1000), FSP1 (Cell Signaling Technology (Danvers, MA, USA), 1:1000) and anti-β-actin (GeneTex, GTX629630, 1:5000). The immune-reactive bands were developed using an enhanced chemiluminescence (ECL) detection system (Pierce Biotechnology, Rockford, IL, USA) and detected using an ImageQuant LAS 400 digital biomolecular imaging system (GE Healthcare Life Sciences, Uppsala, Sweden).

### 2.6. DPP-4 Activity Detection

DPP4 activity Fluorometric assay kit was used for DPP4 activity detection (Biovision, Milpitas, California, CA, USA). Activity was determined in a continuous monitoring assay in a microplate reader (Multiskan Plus MK II, Labsystem) at 405 nm, 37 °C for 30 min under similar conditions. All DPP4 assays were run in duplicates. Serum were diluted in 1:10 and kidney were diluted in 1:200 with DPP4 assay buffer (10 mM Tris-HCl, pH 7.6) and then reaction mixes containing 2 mmol/L substrate (H-Gly-Pro-AMC) were pipetted into each microplate wells. Enzyme activity was expressed in pmol/min/ml (µU/mL).

### 2.7. RNA Isolation and qPCR

Total RNA was isolated from small pieces of kidneys (10–20 mg) using Qiagen RNeasy Mini Kit (Qiagen, Hilden, Germany). Complementary DNA (cDNA) was generated by using the Super script (Invitrogen, Carlsbad, CA). qPCRs were performed in a 7900HT Fast real-time PCR system (Life technologies) using SYBR Green fuorescence with 10 ng of cDNA and quantifed using the delta–delta-cycle threshold (Ct) method(ΔΔCt). All experiments were performed in triplicate and 18S was utilized as an internal control. The used primer sequence for mDPP4 are (Forward 5′-ACCGTGGAAGGTTCTTCTGG and reverse primer sequence 5′-CACAAAGAGTAGGACTTGACC); for αSMA (Forward 5′-CTGACAGAGGCACCACTGAA and reverse 5′-GAAATAGCCAAGCTCAG); for fibronectin (Forward 5′-CGAGGTGACAGAGACCACAA and reverse 5′-CTGGAGTCAAGCCAGACACA). For the experiments in HMVECs, the used primers for hDPP-4 are (Forward 5′-GCACGGCAACACATTGAA and reverse 5′-TGAGGTTCTGAAGGCCTAAATC); for polyoligopeptidase (Forward 5′-CATCTCCCAAGAGGCTGACTA and reverse 5′-GGGCAATAACACAACCAAAGA). These primers were designed by Hokkaido System Science Co. (Hokkaido, Japan).

### 2.8. RNA Extraction and microRNA Array Analysis

Frozen kidney tissues were first placed on the RNA later○R-ICE (Life technologies) for 16 h at −20 °C before the subsequent homogenization process to avoid RNA degradation while extracting high-quality microRNA. Total RNA was isolated using the miRNeasy Kit (Qiagen, Hilden, Germany) following the manufacturer’s instructions. The RNA was quantified with quantified with a nanodrop spectrophotometer (ND-1000, Nano drop Technologies, Wilmington, DE, USA). Ratio of OD 260/280 were between 1.9 and 2.0. The integrity of RNA samples was determined in a bioanalyzer 2100 (Agilent, Santa Clara, CA, USA) and all samples gad RIN values in the range of 8.0–8.5.

Quality-confirmed total RNA samples were assayed and qualified in duplicate using the microRNA microarray. The input for the Agilent microRNA labeling system was 100 ng total RNA. Dephosphorylated and denatured total RNA was labeled with cyanine 3-pCp and subsequently hybridized to the Agilent mouse microRNA microarray release version 15 using the microRNA Complete Labeling and Hyb Kit (Agilent Technologies, Inc., Santa Clara CA). Following hybridization for 20 h, the slides were washed with Gene Expression Wash Buffer Kit (Agilent, Santa Clara, CA, USA) and measured using an Agilent Scanner G2565BA. Agilent Feature Extraction Software version 9.5.1 and GeneSpring GX software version 12.5 (Agilent) were used for data processing, analysis, and monitoring. Predicted targets as extracted from miRanda, TargetScan and PicTar were used to identify the potential putative targets of microRNAs [22]. The common targets predicted by all the three tools were analyzed for the cellular pathways that they enrich using PANTHER BIOLOGICAL CLASSIFICATION databases that categorize a set of genes from the input genes lists on the basis of annotation similarity and then map them as significantly over-represented in a biological pathway [22]. These suggest that the enriched pathways might play a role in the physiological condition being considered. 

### 2.9. RNA Isolation and qPCR 

The complementary DNA was generated by a miScript II RT kit (Qiagen) using the hiSpec buffer method. microRNA expression was quantified using miScript SYBR Green PCR Kit (Qiagen) using 3 ng of complementary DNA. The primers to quantify Mm_miR-29a, Mm_miR-29b, and Mm_miR-29c were the miScript primer assays pre-designed by Qiagen. The mature microRNA sequences were 5′ UAGCACCAUCUGAAAUCGGUUA for Mm_miR-29a, 5′ UAGCACCAUUUGAAAUCAGUGUU for Mm_miR-29b, and 5′ UAGCACCAUUUGAAAUCGGUUA for Mm_miR-29c. All experiments were performed in triplicate, and Hs_RNU6-2_1 (Qiagen) was utilized as an internal control. The primers to quantify Mm_miR-let-7b-1, Mm_miR-let-7b-2, Mm_miR-let-7c, Mm_miR-let-7f, Mm_miR-let-7g, Mm_miR-leti were designed by Qiagen. The mature sequences were UGGAAGACUUGUGAUUUUGUUGU for Mm_miR-let-7b-1, CAACAAGUCACAGCCAGCCUCA for Mm_miR-let-7b-2, CUGUACAACCUUCUAGCUUUCC for Mm_miR-let-7c, CUAUACAAUCUAUUGCCUUCCC for Mm_miR-let-7f, ACUGUACAGGCCACUGCCUUGC for Mm_miR-let-7g and CUGCGCAAGCUACUGCCUUGCU for Mm_miR-let-7i respectively.

### 2.10. Transfection

For the transfection studies, HMVECs, which were maintained in EBM-2 medium supplemented with EGMTM-2 (Lonza, USA), were passaged in 6-well plates with non-proliferative medium (HuMedia-MVG and RPMI at a ratio of 1:3). The HMVECs were transfected with 100 nM of antagomir for miR-29a (Qiagen), using Lipofectamine 2000 transfection reagent (Invitrogen, Carlsbad, CA, USA). The antagomir of miR-let-7b were purchased from Invitrogen, (Carlsbad, CA, USA). The transfection studies of anti-miR-let-7b in the HMVECs at 100 nM concentration was also performed using Lipofectamine 2000 transfection reagent. The cells were incubated for 6 h with lipofectamine and the anti-microRNA complex in antibiotic-free medium, after which the medium was replaced with fresh medium before the cells were incubated for another 48 h. Upon the termination of the incubation, total RNA was isolated using the miRNeasy Kit (Qiagen) following the manufacturer’s instructions.

### 2.11. Statistical Analysis

The data are expressed as the means ± s.e.m. The one-way Anova Tukey test was performed to analyze significance, which was defined as *p* < 0.05, if not specifically mentioned. The post hoc tests were run only if F achieved *p* < 0.05 and there was no significant variance inhomogeneity. In each experiment, N represents the number of separate experiments (in vitro) and the number of mice (in vivo). Technical replicates were used to ensure the reliability of single values. GraphPad Prism software (Ver 5.0f, San Diego, CA, USA) was used for the statistical analysis.

## 3. Results 

### 3.1. Inhibition of ACE Suppresses DPP-4 and Associated TGFβ Signaling in Diabetic Kidneys 

**Figure 1:**
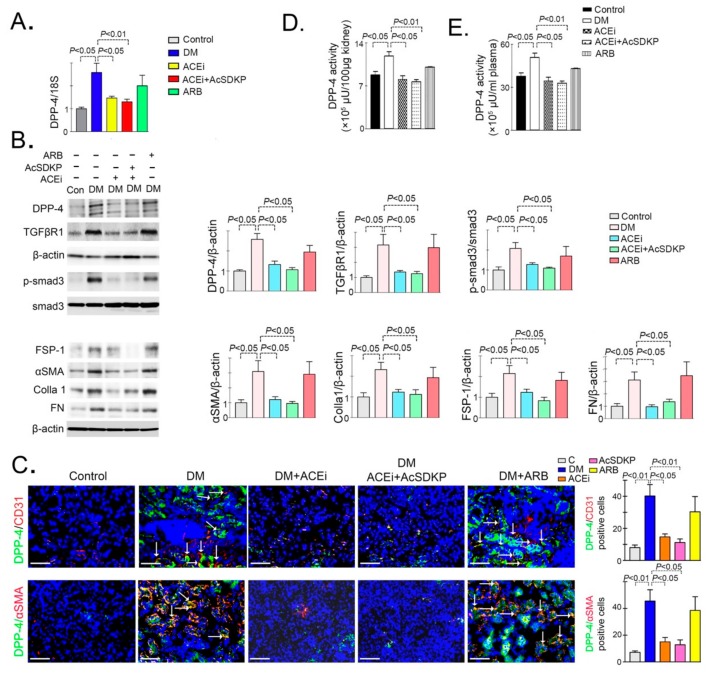
Inhibition of ACE suppresses DPP-4 and associated TGFβ signaling in diabetic kidneys (**A**) Quantitative analysis of DPP-4 mRNA expression by real time PCR using specific primers in the kidney of control, DM, DM+ACEi, DM+combination and DM+ARB treated mice. *N* = 6 were analyzed in each group. 18S was used as internal control to normalize the expression data. (**B**) Western blot analysis of DPP-4, TGFβR1, p-smad3, smad3, FSP-1, αSMA, Colla1a and fibronectin (FN) in the kidney of control, DM, DM + ACEi, DM + combination treatment and DM + ARB treated diabetic mice. Representative blots are shown. Quantification of DPP-4, TGFβR1, smad3 phosphorylation, FSP-1, αSMA, Colla1a and FN by densitometry. The data were normalized by β-actin. *N* = 5 were analyzed in each group. (**C**) Co-immunofloroscence analysis of DPP-4/CD31 and DPP-4/ αSMA in the kidney of control, DM, DM + ACEi, DM + ACEi + AcSDKP and DM + ARB, the representative pictures are shown. Scale bar 50 µm. DPP-4 FITC (green) labeled whereas, CD31 and αSMA are rhodamine labeled and DAPI blue. *N* = 5 were analyzed in each group. (**D**) DPP activity analysis by fluorimeter in kidney homogenate of control, DM, DM + ACEi, DM + combination and DM + ARB treated mice. *N* = 6 were analyzed in each group. (**E**) DPP activity analysis in the plasma of control, DM, DM + ACEi, DM + combination and DM + ARB treated mice. *N* = 6 were analyzed in each group. Data in the graph are presented as mean ± SEM. One-way Anova Tukey test was performed for calculation of statistical significance. C = control (non-diabetic), DM = diabetic group, combination = (ACEi + AcSDKP), Colla1 = collagen I, FN = fibronectin.

Streptozotocin (STZ) induced diabetic CD-1 mouse is a validated model of diabetic kidney disease [28,29]. It has been shown that DPP-4 protein is found higher in the kidneys of diabetic CD-1 mice as compared to kidneys of non-diabetic mice [11]. qPCR analysis showed up-regulated expression of DPP-4 mRNA in the diabetic kidneys, which was reduced by ACEi and combination treatment but not affected by ARB (Figure 1A). Western blot analysis revealed higher protein levels of DPP-4, TGFβR1, smad3 phosphorylation, fibroblast specific protein 1 (FSP-1), αSMA, collagen I and fibronectin in the kidneys of diabetic mice (Figure 1B). Moreover, we observed remarkable higher immuno-labeled CD31/DPP-4 positive cells DPP-4/αSMA in kidneys of diabetic mice as compared to kidneys of non-diabetic control mice (Figure 1C). ACEi and combination treatment significantly reduced the protein level of DPP-4, TGFβR1, smad3 phosphorylation, FSP-1, αSMA, collagen I and fibronectin (Figure 1B); suppressed the higher expression level of CD31/DPP-4 and DPP-4/ αSMA positive cells in the kidneys of diabetic mice (Figure 1C). Whereas, ARB treatment did not affect any significant alteration in the kidneys of diabetic mice (Figure 1B–C). ACEi significantly downregulated gene expression of ACE mRNA whereas, ARB suppressed the gene expression level of AT1R in the kidneys of diabetic mice (Figure S1). 

Furthermore, to test the contribution of DPP-4 in the diabetic nephropathy, we measured the DPP-4 activity in the plasma and the kidney. We observed higher DPP-4 activity level in the kidneys of diabetic mice which was reduced by ACEi and combination treatment whereas not affected by ARB treatment (Figure 1D–E). To study the correlation between the DPP-4 and AcSDKP level, we measured the AcSDKP level in the DPP-4 inhibitor (linagliptin) treated diabetic mice. DPP-4 inhibitor (linagliptin) treatment elevated the level of urine AcSDKP whereas, did not cause any remarkable alteration on the ACE enzyme activity in the kidneys (Figure S2A–B). Taken together, the above results suggest that the ACEi attenuates the DPP-4 mediated renal fibrosis and is associated with AcSDKP-mediated antifibrotic mechanisms.

Comprehensive miRome analysis revealed up-regulated expression of miR-29 and miR-let-7 family members in the kidneys of ACE inhibitor or combination treated diabetic mice

**Figure 2:**
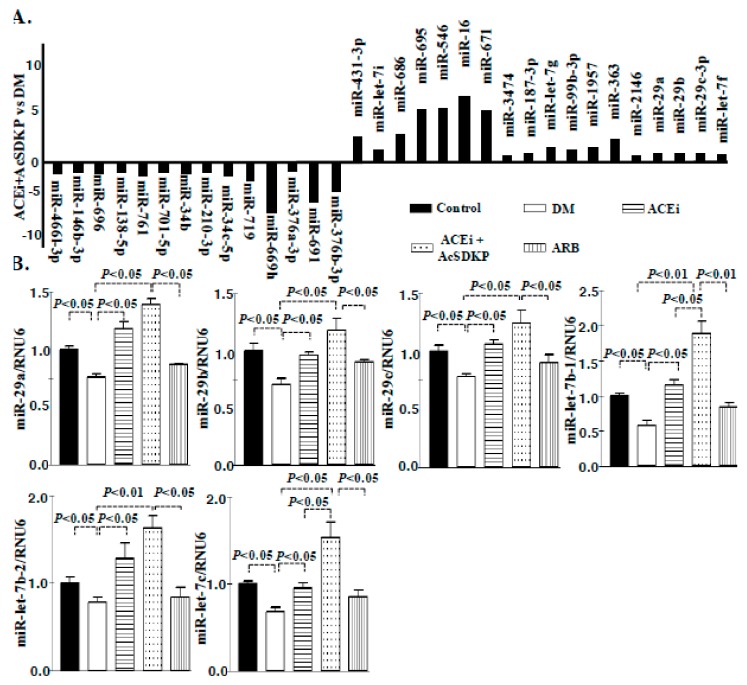
miRome analysis reveal up-regulated expression of miR-29 and miR-let-7 family members in the kidneys of ACE inhibitor or combination treated diabetic mice. (**A**) microRNA-array analysis in the diabetic group vs ACEi + AcSDKP treatment in diabetic mice revealed alteration in the expression level of pro and antifibrotic microRNAs. *N* = 3 were analyzed in each group. (**B**) miR-29 and miR-let-7 family members emerged as important regulatory antifibrotic molecules and validation by the real time PCR using specific primers in the kidney of control, DM, ACEi, combination treatment and ARB group. *N* = 6 were analyzed in each group. Hs_RNU6 was used as internal control to normalize the expression data. Data in the graph are presented as mean±SEM. One-way Anova Tukey test was performed for calculation of statistical significance.

To evaluate miRNA expression signature in the fibrotic kidney of streptozotocin-induced diabetic mice, we performed miRNA microarray analysis. The kidney of control, diabetic, ACEi and combination treated diabetic CD-1 mice were analyzed. A total of 71 microRNAs were differentially expressed; 36 microRNAs were significantly down-regulated whereas 35 microRNAs were significantly up-regulated in the kidneys of diabetic mice. The combination treatment in diabetic mice showed 32 microRNAs restored. Of these 18 microRNAs were restored by up-regulation while 14 were by down-regulation (Figure 2A). Predicted targets of all these altered microRNAs were identified using TargetScan, miRanda, PicTar and miRNAs that had targets predicted by one or more algorithms are shown in (Figure S3). MiR-let-7 (7g, 7i, 7f) and miR-29 (a, b and c) family members were major antifibrotic microRNA and were selected for further study. Since prediction algorithms use specific criteria for target prediction and might be prone to generation of false positive, we followed a common procedure of prioritizing on the miRNAs that occupy the intersection area to increase the confidence of subsequent validation and functional relevance. We then performed the pathway analysis of these common predicted targets using Panther biological classification mapping databases. These tools assign significant over-representation of pathways using annotation-based enrichment of genes from an input list versus those are represented in the whole genome. miR-29 targets map into different ways [22]. The significant (*p* < 0.001) and precise biological processes of the predicted targets are shown in (Table S1A–B; Table S2A–B). A total of 39 biological processes emerged as being significantly over-represented by the predicted target list of miR-29 however, total 37 biological processes over-represented by miR-let-7 (Table S1A-B; Table S2A-B). A considerable number of genes (are listed in the table) categorized into the biological processes (Table S1A–B; Table S2A–B). Importantly among the pathways represented by the targets of miR-29 and miR-let-7 family include TGF-β pathway, integrin signaling pathway and DPP-4 pathway etc (Table S1A-B and Table S2A–B). We validated the microRNA array data of miR-29 and miR-let-7 by qPCR. The qPCR analysis revealed that the kidneys of diabetic mice showed suppressed level of miR-29 and miR-let-7 family members which was up-regulated by ACEi and combination however, did not affected by ARB treatment (Figure 2B; Figure S4). The effect of combination treatment was remarkably more than ACEi (Figure 2B).

### 3.2. Inhibition of ACE Inhibits DPP-4 Level and Associated TGFβ Signaling in Endothelial Cells

**Figure 3:**
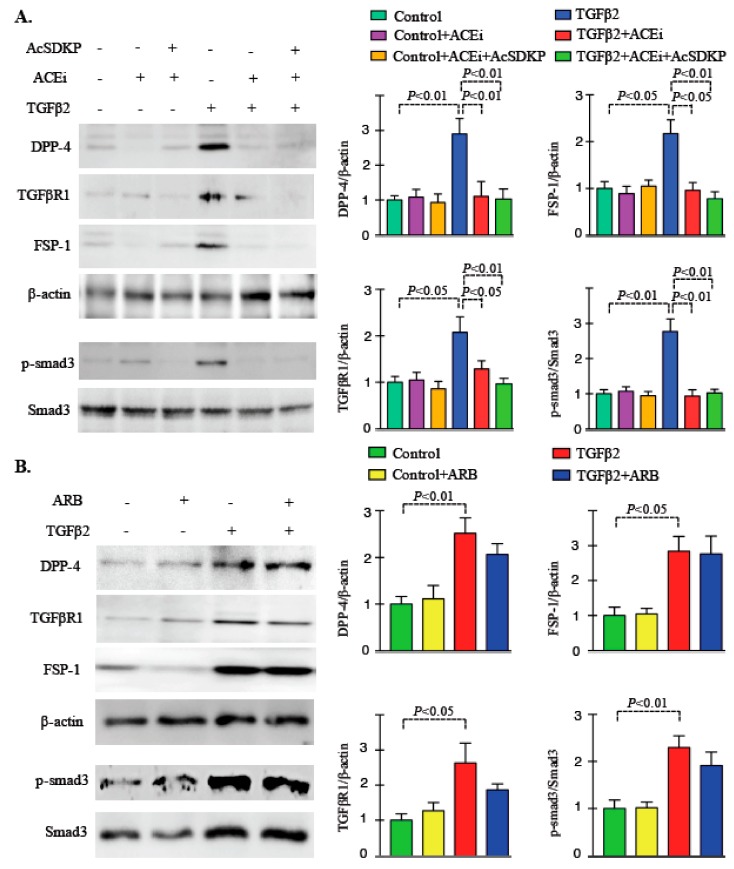
Inhibition of ACE inhibit DPP-4 level and TGFβ signaling in endothelial cells. (**A**) Western blot analysis in the ACEi and ACEi+AcSDKP treated HMVECs in presence and absence of TGFβ2. Quantification of DPP-4, TGFβR1, FSP-1, p-smad3 and smad3 respectively by densitometry. Representative blots are shown. The data was normalized by β-actin. *N* = 3 were analyzed in each group. (**B**) Western blot analysis in the ARB treated HMVECs in presence and absence of TGFβ2. Quantification of DPP-4, TGFβR1, FSP-1, p-smad3 and smad3 respectively by densitometry. Representative blots are shown. The data was normalized by β-actin. *N* = 3 were analyzed in each group. Data in the graph are presented as mean ± SEM. One-way Anova Tukey test was performed for calculation of statistical significance.

In the HMVECs cells, the TGFβ2 treatment caused increase in the protein level of DPP-4, TGFβR1, FSP-1 and smad3 phosphorylation; ACEi and combination treatment decreased the elevated protein level (Figure 3A), suggesting that inhibition of ACE prevented TGFβ2-induced endothelial-to-mesenchymal transition by inhibiting DPP-4 and TGFβ signaling in HMVECs. Whereas, ARB did not exert such effects (Figure 3B), suggesting that ARB has minimal or no effect on TGFβ2 associated gain of fibrogenic characteristics. Moreover, Ang II stimulation in high glucose treated cultured HMVECs, caused upregulation in the gene expression level of SMA and fibronectin (Figure S5). 

### 3.3. Inhibition of ACE Restored the TGFβ2-Associated Disruption of Cross-Talk Regulation between miR-29 and miR-let-7 Family Members in Endothelial Cells

**Figure 4:**
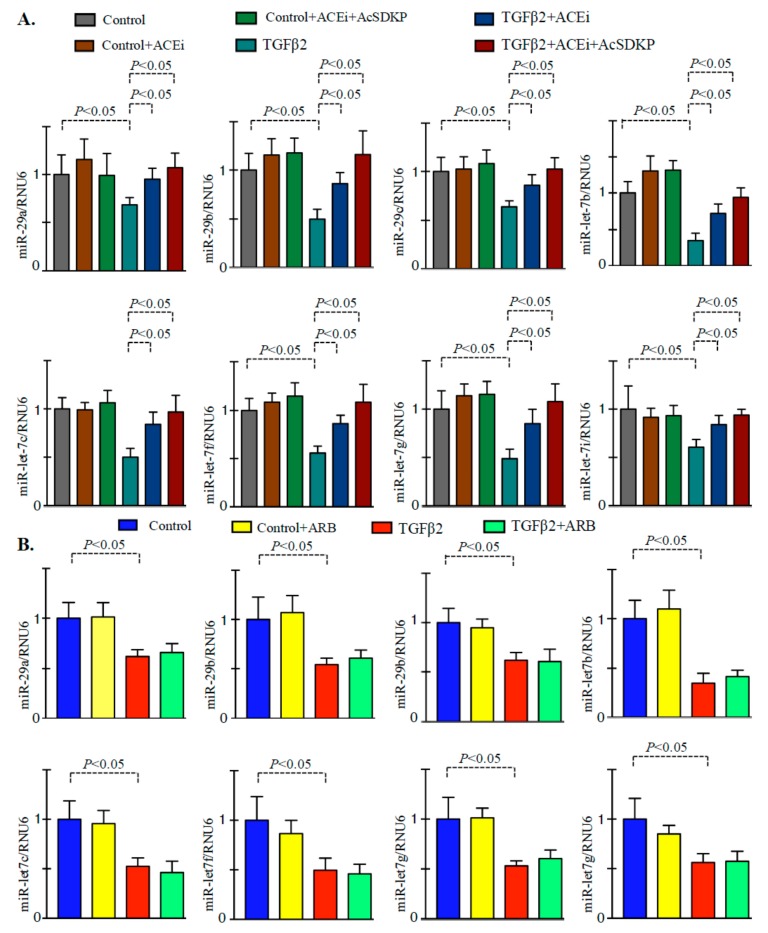
ACEi and combination treatment restore the downregulated level of miR-29 and miR-let-7 family members in the TGFβ2-stimulated HMVECs. (**A**) qPCR analysis of miR-29 and miR-let-7 family members in the control, ACEi, and ACEi+AcSDKP in the presence and absence of TGFβ2 in the HMVECs. *N* = 4 were analyzed in each group. Hs_RNU6 was used as internal control to normalize the expression data. (**B**) qPCR analysis of miR-29 and miR-let-7 family members in the control and ARB stimulation in the presence and absence of TGFβ2. *N* = 4 were analyzed in each group. Hs_RNU6 was used as internal control to normalize the expression data. Data in the graph are presented as mean±SEM. One-way Anova Tukey test was performed for calculation of statistical significance.

**Figure 5:**
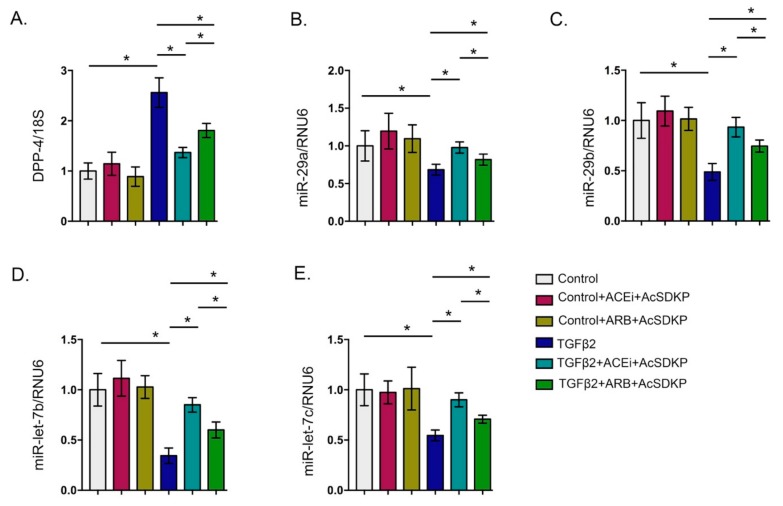
Gene expression analysis of DPP-4 and antifibrotic microRNAs in the combination treatments (ACEi + AcSDKP and ARB + AcSDKP). (**A**) Gene expression analysis of DPP-4 mRNA. (**B**–**C**) Gene expression analysis of miR-29 family members and miR-let-7 family member in the TGFβ2-stimulated HMVECs. *N* = 5 were analyzed in each group Data in the graph are presented as mean±SEM. One-way Anova Tukey test was performed for calculation of statistical significance.

Treatment of TGFβ2 in the HMVECs caused decline in the gene expression level of miR-29 and miR-let-7 family members (Figure 4A). Treatment of the ACEi and combination markedly increased the gene expression level of miR-29 and miR-let-7 family members (Figure 4A). However, we did not observe any remarkable up-regulation in the expression level in the ARB treated TGFβ2-stimulated HMVECs when compared to vehicle-treated TGFβ2 stimulated HMVECs (Figure 4B). Moreover, the effect of ACEi + AcSDKP was more effective in lowering TGFβ2-induced DPP-4 level and ability to restore the TGFβ2-induced suppressed level of miR-29 and miR-let-7 in the HMVECs when compared to ARB + AcSDKP (Figure 5). Furthermore, to analyze the impact of AcSDKP in the selected antifibrotic microRNAs, we used a potent chemical inhibitor of polyoligopeptidase enzyme (POPi-S17092) [30]. Polyoligopeptidase (POP) enzyme catalyzes the synthesis of AcSDKP and POP inhibitor blocks the AcSDKP synthesis in vitro and in vivo [30]. Our results demonstrate that POPi significantly downregulates the gene expression level of POP mRNA; upregulated DPP-4 expression level and suppressed miR-29 and miR-let-7 expression level in the TGFβ2-stimulated HMVECs (Figure S6). 

**Figure 6:**
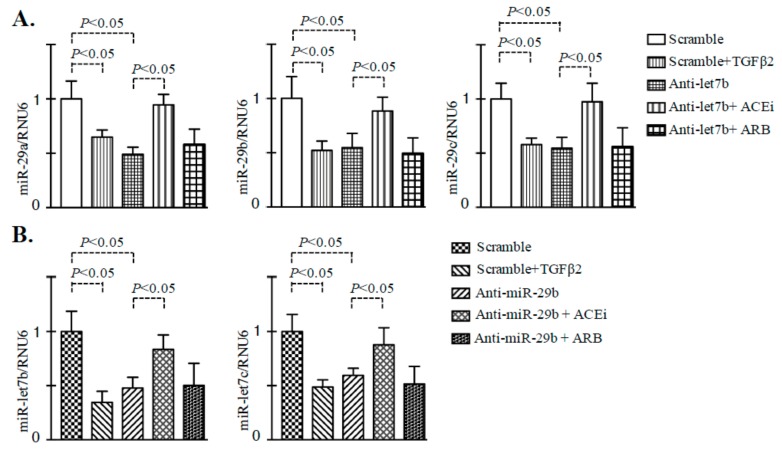
Inhibition of ACE restored the TGFβ2-associated disruption of cross-talk regulation between miR-29 and miR-let-7 family members in the endothelial cells. (**A**) Gene expression analysis of miR-29 family members in the anti-miR-let-7b transfected HMVECs, ACEi+anti-miR-let-7b transfected, and ARB+anti-miR-let-7b transfected HMVECs. *N* = 4 were analyzed in each group. (**B**) Gene expression studies of miR-let-7b and miR-let-7c in the anti-miR-29b transfected HMVECs, ACEi+anti-miR-29b transfected, and ARB+anti-miR-29b transfected HMVECs. *N* = 4 were analyzed in each group. Data in the graph are presented as mean ± SEM. One-way Anova Tukey test was performed for calculation of statistical significance.

In the present study, we analyzed the effect of ACEi and ARB on the homeostasis of anti-fibrotic microRNAs crosstalk in the endothelial cells. Inhibition of the miR-let-7b by antagomir (anti-miR-let-7b) transfection displayed remarkable reduction in the expression level of miR-29a, miR-29b and miR-29c (Figure 6A); inhibition of ACE in such anti-miR-let-7b transfected HMVECs caused significant restoration in the expression level of miR-29a, miR-29b and miR-29c. Similarly, inhibition of miR-29b by antagomir (anti-miR-29b) transfection caused remarkable decline in the expression level of miR-let-7b and miR-let-7c (Figure 6B); inhibition of ACE in such anti-miR-miR-29b transfected HMVECs caused significant restoration in the expression level of miR-let-7b and miR-let-7c. However, we did not observe any remarkably difference in the expression level of miR-29 and miR-let-7 family members in the ARB-treated anti-miR-let-7b or in anti-miR-29b transfected HMVECs. 

## 4. Discussion 

In the present study, we show in a preclinical setting of clinical relevance that inhibition of ACE is one of the therapy for renal fibrosis which is either directly associated with the suppression of pathobiological mechanisms of DPP-4 and restoration of antifibrotic microRNAs or indirectly by AcSDKP-mediated-restoration of anti-fibrotic microRNAs in kidney whereas treatment of ARB is unable to show such protective effects in the mouse model of diabetic kidney disease. We investigated the comparative effects of the RAAS inhibitors, such as angiotensin-converting enzyme inhibitors (ACEi) and angiotensin receptor blockers (ARB) on the DPP-4-associated mesenchymal activation in diabetic kidney and its implication in renal fibrosis. Our results demonstrate that inhibition of ACE protects renal fibrosis by suppressing DPP-4-associated mesenchymal transformations and elevating the gene expression of antifibrotic microRNAs in the kidneys of diabetic mice. From the results, it is clear that ACEi-mediated renal protection is due to its ability to activate the AcSDKP-associated renal protections whereas, ARB did not show such effects since, ARB failed to induced the AcSDKP level in diabetic mice. 

Meta-analysis studies showing that ACEi alone or in combination with ARB, are the most effective strategy for reducing the risk of progression to ESRD in diabetic nephropathy. Few studies have shown the comparative effect of ACEi and ARB on urinary protein excretion in experimental and human type 1 nephropathy [6,31,32,33]. However, combination therapies (ARB + ACEi) may have diverse effects on reno-protection, in spite of remarkable reduction in the albuminuria [34,35,36,37]. The studies regarding the combined therapy of ACEi + ARB is not matter of discussion in the present study. However, the safe use and comparative antifibrotic nature of ACEi and ARB are the matter of discussion in the present study. Previous studies in the diabetic and non-diabetic subjects have shown differential effects in terms of renal function and protection [5,8,38,39,40]. However, some studies have been shown for better effects of ACEi over ARB on renal protections in the diabetic patients who have symptoms of chronic kidney disease [5,8,38]. Some of clinical trials have shown protective nature of ARB in kidney outcome, however it is also true that there is not much clinical trial that clearly demonstrated the significant differences between ACEi and ARB in renal outcome in diabetes. However even though such limitation, Mauer et al. from Minnesota analyzed for five years ARB losartan or ACEi enalapril (compared to placebo) use in normotensive type 1 diabetic patients without albuminuria and found losartan use was associated with significant increase in the onset of microalbuminuria when compared to placebo; enalapril use was associated with insignificant suppression in the onset of microalbuminuria when compared to placebo [33]. Furthermore, Mauer et al., performed renal biopsies before and after the drug intervention and found only losartan group trended increased in glomerular mesangial matrix fractions in post-intervention biopsy samples but not in the pre-intervention samples (*n* = 50 in each group, *p* = 0.07) [33], suggested the important difference between ARB and ACEi and superiority in ACEi [41].

Inhibition of ACE suppressed angiotensin II formation, thus, less angiotensin II binding to the AT1 receptor and AT2 receptor [42]. When ARB is used, that blocked AT1 receptor signaling, hence, angiotensin II accumulates [43]. Accumulated angiotensin II binds and further induce the AT2 receptor signaling. However, the results of AT2 receptor activation could be organ protective [44,45,46,47] but few reports suggest the harmful for organ protection [43]. Our results, suggests that such accumulation of angiotensin II would not show protective effect. Moreover, the significance of angiotensin II accumulation and AT2 receptor activation in organ protection needs further investigation.

Antifibrotic mechanism of ACE inhibition is associated with the induced level of AcSDKP, reduction in the DPP-4 and TGF-β signaling regfulated by miR-29s and miR-let-7s. Inhibition of ACE mediated anti-fibrotic action is physiologically relevant since fibrotic model of diabetic CD-1 mice exhibited suppressed level of AcSDKP, DPP-4 induction, lower level of anti-fibrotic microRNAs whereas, these alterations were not found in non-fibrotic diabetic 129Sv mice [27]. Therefore, suppressed level of AcSDKP is the key pro-fibrotic phenotype in the diabetic kidneys, which is associated suppressed level of anti-fibrotic microRNAs such as miR-29s and miR-let-7s [27]. AcSDKP acts on fibrosis by suppressing the DPP-4 level, and associated TGFβ signaling-linked mesenchymal transformations [9,27,48]. DPP-4 inhibition in the diabetic kidney, was found to ameliorate renal fibrosis which is associated with higher level of AcSDKP in the diabetic mice [11,27]. It is evident from our data that inhibition of ACE is associated with significant suppression in DPP-4, and mitigates higher level of TGF-β signaling and mesenchymal transformations in the TGF-β stimulated cultured endothelial whereas, ARB failed to show protective effects in the cultured cells. This study establishes a new antifibrotic mechanism of ACEi that includes the DPP-4 inhibition in the kidneys of diabetic mice and in the TGF-β stimulated cultured cells. 

**Figure 7:**
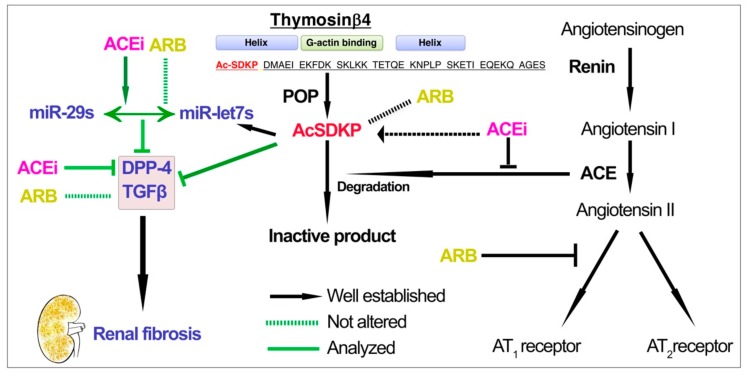
Working hypothesis for ACEi action on the suppression of DPP-4 associated fibrogenic program and restoration of antifibrotic microRNAs.

Recently there are several reports that describe the regulatory role of microRNAs in the diabetic nephropathy [49,50,51,52,53,54,55,56,57,58]. We sought to evaluate the miRNA status in the diabetic nephropathy and to assess the physiological relevance with respect to ACE inhibition in diabetic mice. Our data suggests that miR-29 and miR-let-7 identify as key antifibrotic players. Members of miR-29 family negatively regulate DPP-4, so we tried to find out functional significance in between TGFβRs and DPP-4 with respect to renal fibrosis [11]. In previous studies, TGFβ2 treatment in the HMVECs caused gain of features of myo-fibroblasts and reported to show down-regulated expression level of miR-29 and miR-let-7 [9,11]. MiR-let-7 family members were key anti-fibrotic players targeting TGFβR1 in the HMVECs as evidenced by our results [9]. AcSDKP is the key essential peptide that regulates the antifibrotic crosstalk between miR-29s and miR-let-7s in the endothelial cells and this antifibrotic microRNA crosstalk is critical for its anti-EndMT effect [3,27]. Moreover, inhibition of ACE caused up-regulation of antifibrotic microRNAs (miR-29 and miR-let-7 family members) and restored the antifibrotic cross-talk in the cultured endothelial cells while ARB has minimal effect suggesting that ACEi has a critical anti-EndMT effect. Figure 7 depicts the comparative effect of ACEi and ARB on stimulating the antifibrotic microRNAs by increasing the AcSDKP mediated antifibrotic mechanisms.

Conclusion: In conclusion, these data supporting the idea that inhibition of ACE protects renal fibrosis by its ability to induce the AcSDKP-associated renal protections, either by suppressing DPP-4-associated mesenchymal transformations or by elevating the gene expression of antifibrotic microRNAs in the kidneys of diabetic mice, whereas, ARB did not show such effects. The study adds valuable information in the significance of ACEi and DPP-4 biology in the kidneys of diabetic mice.

## Supplementary Materials

The following are available online at https://www.mdpi.com/2073-4425/11/2/211/s1, Table S1: Determination of biological processes and relevant pathways from predicted targets of miR-29 family members. (A) Biological processes significantly (p < 0.001) enriched in the predicted targets of miR-29 family members. (B) Relevant Biological pathway significantly from the predicted targets of miR-29 family members, Table S2: Determination of biological processes and relevant pathways from predicted targets of miR-let-7 family members. (A) Biological processes significantly (p < 0.001) enriched in the predicted targets of miR-let-7 family members. (B) Relevant Biological pathway significantly from the predicted targets of the miR-let-7 family members, Figure S1: Gene expression analysis of ACE in kidneys of ACE inhibitor treated diabetic mice and gene expression analysis of AT1R in kidneys of ARB treated diabetic mice., Figure S2A: Measurement of urine AcSDKP in the control, DM and linagliptin treated diabetic mice., Figure S2B: ACE activity in the kidneys of control and diabetic, and linagliptin treated diabetic mice., Figure S3: Altered microRNAs were analyzed using online programs (TargetScan, PicTar and miRanda) for target identification., Figure S4: Gene expression level of miR-let-7f, miR-let-7g and miR-let-7i in the kidneys of control, diabetic and ARB treated diabetic mice., Figure S5: Gene expression level of αSMA and fibronectin in the Ang II stimulated high glucose treated HMVECs., Figure S6: Gene expression analysis of DPP-4 and antifibrotic microRNAs in the POP inhibitor treated TGFβ2 stimulated HMVECs.

## References

[1] Held P.J., Port F.K., Webb R.L., Wolfe R.A., Garcia J.R., Blagg C.R., Agodoa L.Y. (1991). The united states renal data system’s 1991 annual data report: An introduction. Am. J. Kidney Dis..

[2] Liu Y. (2011). Cellular and molecular mechanisms of renal fibrosis. Nat. Rev. Nephrol..

[3] Srivastava S.P., Hedayat F.A., Kanasaki K., Goodwin J.E. (2019). Microrna crosstalk influences epithelial-to-mesenchymal, endothelial-to-mesenchymal, and macrophage-to-mesenchymal transitions in the kidney. Front. Pharmacol..

[4] Ganesh J., Viswanathan V. (2011). Management of diabetic hypertensives. Indian J. Endocrinol. Metab..

[5] Hsu F.Y., Lin F.J., Ou H.T., Huang S.H., Wang C.C. (2017). Renoprotective effect of angiotensin-converting enzyme inhibitors and angiotensin ii receptor blockers in diabetic patients with proteinuria. Kidney Blood Press Res..

[6] Palmer S.C., Mavridis D., Navarese E., Craig J.C., Tonelli M., Salanti G., Wiebe N., Ruospo M., Wheeler D.C., Strippoli G.F. (2015). Comparative efficacy and safety of blood pressure-lowering agents in adults with diabetes and kidney disease: A network meta-analysis. Lancet.

[7] Gu J., Yang M., Qi N., Mei S., Chen J., Song S., Jing Y., Chen M., He L., Sun L. (2016). Olmesartan prevents microalbuminuria in db/db diabetic mice through inhibition of angiotensin ii/p38/sirt1-induced podocyte apoptosis. Kidney Blood Press Res..

[8] Laverman G.D., Remuzzi G., Ruggenenti P. (2004). Ace inhibition versus angiotensin receptor blockade: Which is better for renal and cardiovascular protection?. J. Am. Soc. Nephrol..

[9] Nagai T., Kanasaki M., Srivastava S., Nakamura Y., Ishigaki Y., Kitada M., Shi S., Kanasaki K., Koya D. (2014). N-acetyl-seryl-aspartyl-lysyl-proline inhibits diabetes-associated kidney fibrosis and endothelial-mesenchymal transition. Biomed. Res. Int..

[10] Von Websky K., Reichetzeder C., Hocher B. (2014). Physiology and pathophysiology of incretins in the kidney. Curr. Opin. Nephrol. Hypertens..

[11] Kanasaki K., Shi S., Kanasaki M., He J., Nagai T., Nakamura Y., Ishigaki Y., Kitada M., Srivastava S.P., Koya D. (2014). Linagliptin-mediated dpp-4 inhibition ameliorates kidney fibrosis in streptozotocin-induced diabetic mice by inhibiting endothelial-to-mesenchymal transition in a therapeutic regimen. Diabetes.

[12] Xu J., Wang J., Cheng Y., Li X., He M., Zhu J., Han H., Wei G., Kong H., Xie W. (2018). Glucagon-like peptide-1 mediates the protective effect of the dipeptidyl peptidase iv inhibitor on renal fibrosis via reducing the phenotypic conversion of renal microvascular cells in monocrotaline-treated rats. Biomed. Res. Int..

[13] Uchida T., Oda T., Matsubara H., Watanabe A., Takechi H., Oshima N., Sakurai Y., Kumagai H. (2017). Renoprotective effects of a dipeptidyl peptidase 4 inhibitor in a mouse model of progressive renal fibrosis. Ren. Fail.

[14] Min H.S., Kim J.E., Lee M.H., Song H.K., Kang Y.S., Lee M.J., Lee J.E., Kim H.W., Cha J.J., Chung Y.Y. (2014). Dipeptidyl peptidase iv inhibitor protects against renal interstitial fibrosis in a mouse model of ureteral obstruction. Lab Invest..

[15] Shi S., Srivastava S.P., Kanasaki M., He J., Kitada M., Nagai T., Nitta K., Takagi S., Kanasaki K., Koya D. (2015). Interactions of dpp-4 and integrin beta1 influences endothelial-to-mesenchymal transition. Kidney Int..

[16] Kanasaki M., Nagai T., Kitada M., Koya D., Kanasaki K. (2011). Elevation of the anti-fibrotic peptide n-acetyl-seryl-aspartyl-lysyl-proline: A blood pressure-independent beneficial effect of angiotensin i-converting enzyme inhibitors. Fibrogen. Tissue Rep..

[17] Kanasaki K., Koya D., Sugimoto T., Isono M., Kashiwagi A., Haneda M. (2003). N-acetyl-seryl-aspartyl-lysyl-proline inhibits tgf-beta-mediated plasminogen activator inhibitor-1 expression via inhibition of smad pathway in human mesangial cells. J. Am. Soc. Nephrol. JASN.

[18] Shibuya K., Kanasaki K., Isono M., Sato H., Omata M., Sugimoto T., Araki S., Isshiki K., Kashiwagi A., Haneda M. (2005). N-acetyl-seryl-aspartyl-lysyl-proline prevents renal insufficiency and mesangial matrix expansion in diabetic db/db mice. Diabetes.

[19] Omata M., Taniguchi H., Koya D., Kanasaki K., Sho R., Kato Y., Kojima R., Haneda M., Inomata N. (2006). N-acetyl-seryl-aspartyl-lysyl-proline ameliorates the progression of renal dysfunction and fibrosis in wky rats with established anti-glomerular basement membrane nephritis. J. Am. Soc. Nephrol. JASN.

[20] Nitta K., Shi S., Nagai T., Kanasaki M., Kitada M., Srivastava S.P., Haneda M., Kanasaki K., Koya D. (2016). Oral administration of n-acetyl-seryl-aspartyl-lysyl-proline ameliorates kidney disease in both type 1 and type 2 diabetic mice via a therapeutic regimen. Biomed. Res. Int..

[21] Pandey A.K., Verma G., Vig S., Srivastava S., Srivastava A.K., Datta M. (2011). Mir-29a levels are elevated in the db/db mice liver and its overexpression leads to attenuation of insulin action on pepck gene expression in hepg2 cells. Mol. Cell Endocrinol..

[22] Kaur K., Pandey A.K., Srivastava S., Srivastava A.K., Datta M. (2011). Comprehensive mirnome and in silico analyses identify the wnt signaling pathway to be altered in the diabetic liver. Mol. Biosyst..

[23] Nascimento L.R.D., Domingueti C.P. (2019). Micrornas: New biomarkers and promising therapeutic targets for diabetic kidney disease. J. Bras. Nefrol..

[24] Lorente-Cebrian S., Gonzalez-Muniesa P., Milagro F.I., Martinez J.A. (2019). Micrornas and other non-coding rnas in adipose tissue and obesity: Emerging roles as biomarkers and therapeutic targets. Clin. Sci. (Lond).

[25] Dehaini H., Awada H., El-Yazbi A., Zouein F.A., Issa K., Eid A.A., Ibrahim M., Badran A., Baydoun E., Pintus G. (2019). Micrornas as potential pharmaco-targets in ischemia-reperfusion injury compounded by diabetes. Cells.

[26] Zhao X., Chen Z., Zhou Z., Li Y., Wang Y., Zhou Z., Lu H., Sun C., Chu X. (2019). High-throughput sequencing of small rnas and analysis of differentially expressed micrornas associated with high-fat diet-induced hepatic insulin resistance in mice. Genes Nutr..

[27] Srivastava S.P., Shi S., Kanasaki M., Nagai T., Kitada M., He J., Nakamura Y., Ishigaki Y., Kanasaki K., Koya D. (2016). Effect of antifibrotic micrornas crosstalk on the action of n-acetyl-seryl-aspartyl-lysyl-proline in diabetes-related kidney fibrosis. Sci. Rep..

[28] Sugimoto H., Grahovac G., Zeisberg M., Kalluri R. (2007). Renal fibrosis and glomerulosclerosis in a new mouse model of diabetic nephropathy and its regression by bone morphogenic protein-7 and advanced glycation end product inhibitors. Diabetes.

[29] Srivastava S.P., Li J., Kitada M., Fujita H., Yamada Y., Goodwin J.E., Kanasaki K., Koya D. (2018). Sirt3 deficiency leads to induction of abnormal glycolysis in diabetic kidney with fibrosis. Cell Death Dis..

[30] Zhou D., Li B.H., Wang J., Ding Y.N., Dong Y., Chen Y.W., Fan J.G. (2016). Prolyl oligopeptidase inhibition attenuates steatosis in the l02 human liver cell line. PLoS ONE.

[31] Scheen A.J. (2004). Renin-angiotensin system inhibition prevents type 2 diabetes mellitus. Part 1. A meta-analysis of randomised clinical trials. Diabetes Metab..

[32] Jacobsen P., Andersen S., Jensen B.R., Parving H.H. (2003). Additive effect of ace inhibition and angiotensin ii receptor blockade in type i diabetic patients with diabetic nephropathy. J. Am. Soc. Nephrol..

[33] Mauer M., Zinman B., Gardiner R., Suissa S., Sinaiko A., Strand T., Drummond K., Donnelly S., Goodyer P., Gubler M.C. (2009). Renal and retinal effects of enalapril and losartan in type 1 diabetes. N. Eng. J. Med..

[34] Mogensen C.E., Neldam S., Tikkanen I., Oren S., Viskoper R., Watts R.W., Cooper M.E. (2000). Randomised controlled trial of dual blockade of renin-angiotensin system in patients with hypertension, microalbuminuria, and non-insulin dependent diabetes: The candesartan and lisinopril microalbuminuria (calm) study. Bmj.

[35] Mann J.F., Schmieder R.E., McQueen M., Dyal L., Schumacher H., Pogue J., Wang X., Maggioni A., Budaj A., Chaithiraphan S. (2008). Renal outcomes with telmisartan, ramipril, or both, in people at high vascular risk (the ontarget study): A multicentre, randomised, double-blind, controlled trial. Lancet.

[36] Kunz R., Friedrich C., Wolbers M., Mann J.F. (2008). Meta-analysis: Effect of monotherapy and combination therapy with inhibitors of the renin angiotensin system on proteinuria in renal disease. Ann. Intern. Med..

[37] Mauer M., Fioretto P. (2005). Preventing microalbuminuria in type 2 diabetes. N. Eng. J. Med..

[38] Wu H.Y., Peng C.L., Chen P.C., Chiang C.K., Chang C.J., Huang J.W., Peng Y.S., Tu Y.K., Chu T.S., Hung K.Y. (2017). Comparative effectiveness of angiotensin-converting enzyme inhibitors versus angiotensin ii receptor blockers for major renal outcomes in patients with diabetes: A 15-year cohort study. PLoS ONE.

[39] Mavridis D., Palmer S.C., Strippoli G.F. (2016). Comparative superiority of ace inhibitors over angiotensin receptor blockers for people with ckd: Does it matter?. Am. J. Kidney Dis..

[40] Baltatzi M., Savopoulos C., Hatzitolios A. (2011). Role of angiotensin converting enzyme inhibitors and angiotensin receptor blockers in hypertension of chronic kidney disease and renoprotection. Study results. Hippokratia.

[41] Wu H.Y., Huang J.W., Lin H.J., Liao W.C., Peng Y.S., Hung K.Y., Wu K.D., Tu Y.K., Chien K.L. (2013). Comparative effectiveness of renin-angiotensin system blockers and other antihypertensive drugs in patients with diabetes: Systematic review and bayesian network meta-analysis. BMJ.

[42] Wolf G., Ritz E. (2005). Combination therapy with ace inhibitors and angiotensin ii receptor blockers to halt progression of chronic renal disease: Pathophysiology and indications. Kidney Int..

[43] Naito T., Ma L.J., Yang H., Zuo Y., Tang Y., Han J.Y., Kon V., Fogo A.B. (2010). Angiotensin type 2 receptor actions contribute to angiotensin type 1 receptor blocker effects on kidney fibrosis. Am. J. Physiol. Ren. Physiol..

[44] Matavelli L.C., Huang J., Siragy H.M. (2011). Angiotensin at(2) receptor stimulation inhibits early renal inflammation in renovascular hypertension. Hypertension.

[45] Carey R.M., Wang Z.Q., Siragy H.M. (2000). Role of the angiotensin type 2 receptor in the regulation of blood pressure and renal function. Hypertension.

[46] Danyel L.A., Schmerler P., Paulis L., Unger T., Steckelings U.M. (2013). Impact of at2-receptor stimulation on vascular biology, kidney function, and blood pressure. Integr. Blood Press Control.

[47] Padia S.H., Carey R.M. (2013). At2 receptors: Beneficial counter-regulatory role in cardiovascular and renal function. Pflugers Arch..

[48] Li J., Shi S., Srivastava S.P., Kitada M., Nagai T., Nitta K., Kohno M., Kanasaki K., Koya D. (2017). Fgfr1 is critical for the anti-endothelial mesenchymal transition effect of n-acetyl-seryl-aspartyl-lysyl-proline via induction of the map4k4 pathway. Cell Death Dis..

[49] Wang B., Jha J.C., Hagiwara S., McClelland A.D., Jandeleit-Dahm K., Thomas M.C., Cooper M.E., Kantharidis P. (2013). Transforming growth factor-beta1-mediated renal fibrosis is dependent on the regulation of transforming growth factor receptor 1 expression by let-7b. Kidney Int..

[50] Macconi D., Tomasoni S., Romagnani P., Trionfini P., Sangalli F., Mazzinghi B., Rizzo P., Lazzeri E., Abbate M., Remuzzi G. (2012). Microrna-324-3p promotes renal fibrosis and is a target of ace inhibition. J. Am. Soc. Nephrol..

[51] Srivastava S.P., Shi S., Koya D., Kanasaki K. (2014). Lipid mediators in diabetic nephropathy. Fibrog. Tissue Rep..

[52] Srivastava S.P., Koya D., Kanasaki K. (2013). Micrornas in kidney fibrosis and diabetic nephropathy: Roles on emt and endmt. Biomed. Res. Int..

[53] Liu Y., Li H., Liu J., Han P., Li X., Bai H., Zhang C., Sun X., Teng Y., Zhang Y. (2017). Variations in microrna-25 expression influence the severity of diabetic kidney disease. J. Am. Soc. Nephrol..

[54] Zhou Z., Wan J., Hou X., Geng J., Li X., Bai X. (2017). Microrna-27a promotes podocyte injury via ppargamma-mediated beta-catenin activation in diabetic nephropathy. Cell Death Dis..

[55] Lee H.W., Khan S.Q., Khaliqdina S., Altintas M.M., Grahammer F., Zhao J.L., Koh K.H., Tardi N.J., Faridi M.H., Geraghty T. (2017). Absence of mir-146a in podocytes increases risk of diabetic glomerulopathy via up-regulation of erbb4 and notch-1. J. Biol. Chem..

[56] Wang B., Koh P., Winbanks C., Coughlan M.T., McClelland A., Watson A., Jandeleit-Dahm K., Burns W.C., Thomas M.C., Cooper M.E. (2011). Mir-200a prevents renal fibrogenesis through repression of tgf-beta2 expression. Diabetes.

[57] Chen H.Y., Zhong X., Huang X.R., Meng X.M., You Y., Chung A.C., Lan H.Y. (2014). Microrna-29b inhibits diabetic nephropathy in db/db mice. Mol. Ther..

[58] Krupa A., Jenkins R., Luo D.D., Lewis A., Phillips A., Fraser D. (2010). Loss of microrna-192 promotes fibrogenesis in diabetic nephropathy. J. Am. Soc. Nephrol..

