# Supplementary material for: Inhibition of Angiotensin-Converting Enzyme Ameliorates Renal Fibrosis by Mitigating DPP-4 Level and Restoring Antifibrotic MicroRNAs
Source: Genes (Basel). 2020 Feb 18;11(2):211. doi: 10.3390/genes11020211 (PMC7074526; doi:10.3390/genes11020211)
Supplement: Supplementary file 1 [file genes-11-00211-s001.pdf]

Supplementary information

Inhibition of angiotensin-converting enzyme ameliorates renal fibrosis by mitigating DPP-4 level and restoring antifibrotic microRNAs

Swayam Prakash Srivastava[1,2], Julie E. Goodwin[2], Keizo Kanasaki[1,3,4], Daisuke Koya[1,3]

[1]Department of Diabetology & Endocrinology, Kanazawa Medical University, Uchinada, Ishikawa, Japan 920-0293

[2]Department of Pediatrics Yale University School of Medicine, New Haven, CT, 06520, USA

[3]Division of Anticipatory Molecular Food Science and Technology, Kanazawa Medical University, Uchinada, Ishikawa, Japan 920-0293

[4]Shimane University Faculty of Medicine, Internal Medicine 1, Enya-cho, Izumo, Shimane 693-8501, Japan

***: Co-correspondence**

Address correspondence to:

Keizo Kanasaki,

E-mail: kkanasak@kanazawa-med.ac.jp,

Or

Daisuke Koya

E-mail: koya0516@kanazawa-med.ac.jp,

Department of Diabetology & Endocrinology

Kanazawa Medical University

Uchinada, Ishikawa 920-0293, Japan

TEL: 81-76-286-2211(Ex3305)

FAX: 81-76-286-6927

Figure S1: Gene expression analysis of ACE in kidneys of ACE inhibitor treated diabetic mice and gene expression analysis of AT1R in kidneys of ARB treated diabetic mice

Real time PCR analysis using specific primers. Five number of mice were analyzed in each group. 18S was used to normalize the expression data. Data in the graph are presented as mean±SEM. One way Anova Tukey test was performed for calculation of statistical significance.

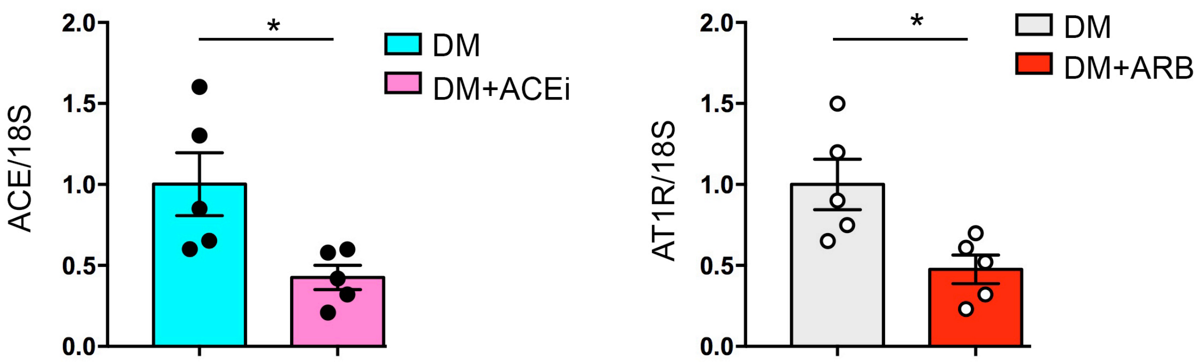

Supplementary Fig 2A. Measurement of urine AcSDKP in the control, DM and linagliptin treated diabetic mice. N=4 were analyzed. Data in the graph are presented as mean±SEM
Supplementary Fig. 2B. ACE activity in the kidneys of control and diabetic, and linagliptin treated diabetic mice. N=4 were analyzed. Data in the graph are presented as mean±SEM. One way Anova Tukey test was performed for calculation of statistical significance.

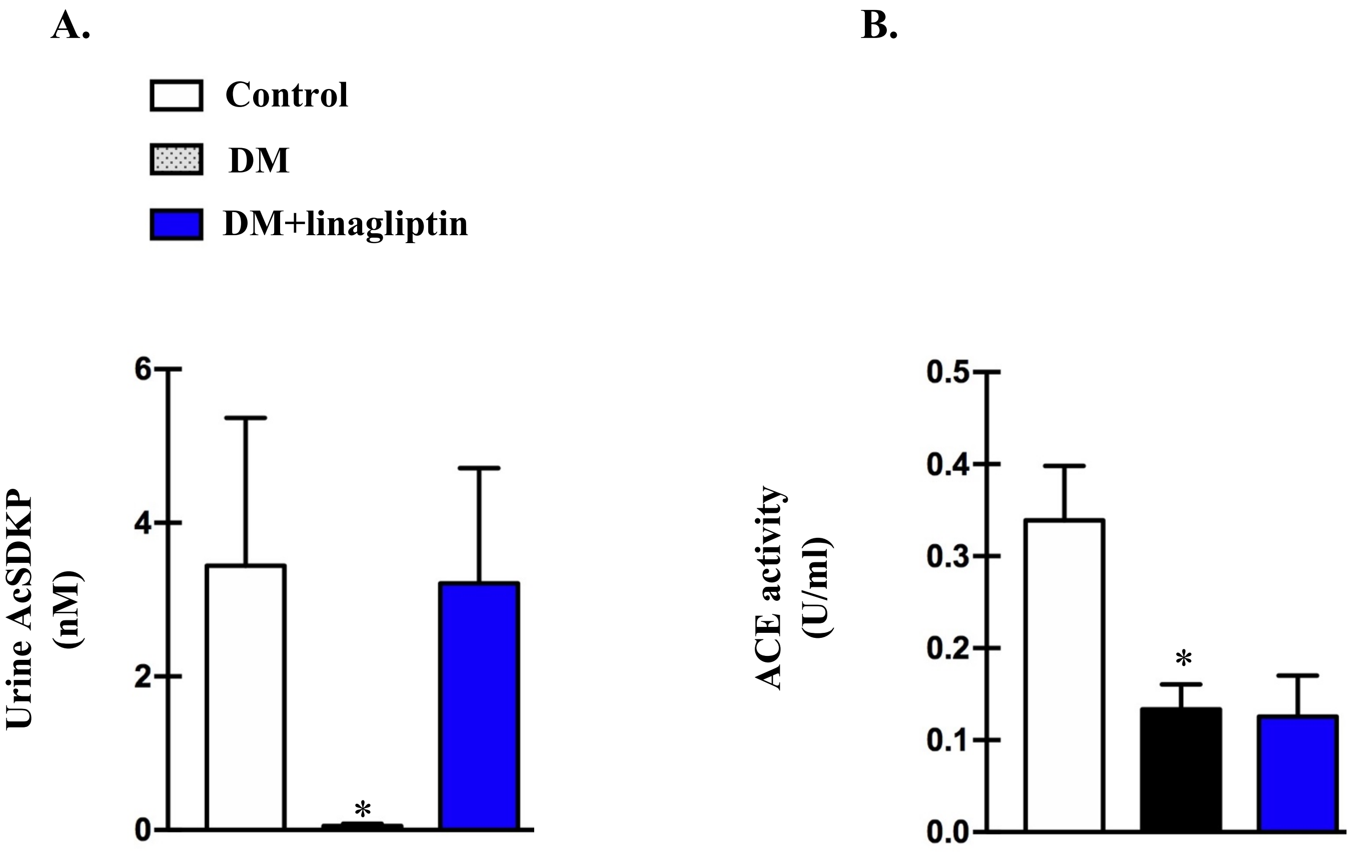

Figure S3 Altered microRNAs were analyzed using online programs (TargetScan, PicTar and miRanda) for target identification.

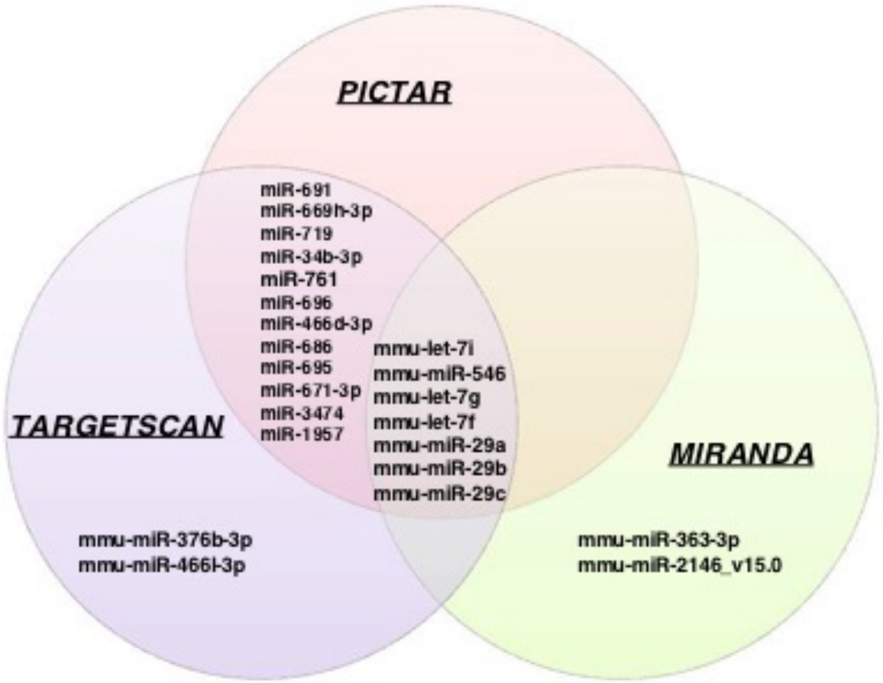

Table S1. Determination of biological processes and relevant pathways from predicted targets of miR-29 family members.

Table S1A: Biological processes significantly (p<0.001) enriched in the predicted targets of miR-29 family members

S.N.	Biological process	Number of genes from our predicted target list
1	cellular process	513
2	developmental process	286
3	regulation of liquid surface tension	29
4	biological regulation	302
5	cell communication	310
6	transcription, DNA-dependent	197
7	transcription from RNA polymerase II promoter	195
8	anatomical structure morphogenesis	94
9	RNA metabolic process	230
10	cellular component morphogenesis	87
11	regulation of transcription from RNA polymerase II promoter	155
12	regulation of biological process	203
13	homeostatic process	35
14	regulation of nucleobase-containing compound metabolic process	161
15	cell-cell adhesion	70
16	cellular component organization	117
17	cell adhesion	101
18	biological adhesion	101
19	ectoderm development	101
20	nucleobase-containing compound metabolic process	282
21	cellular component organization or biogenesis	118
22	receptor-mediated endocytosis	40
23	endocytosis	59
24	vesicle-mediated transport	100
25	metabolic process	596
26	protein transport	128
27	primary metabolic process	511
28	blood circulation	30
29	macrophage activation	44
30	response to pheromone	0

31	intracellular protein transport	123
32	mesoderm development	103
33	system development	141
34	multicellular organismal process	168
35	single-multicellular organism process	168
36	localization	212
37	gamete generation	57
38	transport	203
39	cell-matrix adhesion	20
40	system process	139
41	protein phosphorylation	58
42	spermatogenesis	30
43	cell cycle	120
44	reproduction	62
45	cell-cell signaling	74
46	cellular protein modification process	101
47	synaptic transmission	42
48	negative regulation of apoptotic process	24
49	nervous system development	80

Table S1B: Relevant Biological pathway significantly from the predicted targets of miR-29 family members

S.N.	Biological pathway
1	TGF-beta signaling pathway
2	FGF signaling pathway
3	Hypoxia response via HIF activation
4	Integrin signalling pathway
5	DPP-4 pathway

6	Ras Pathway
7	Apoptosis signaling pathway
8	PI3 kinase pathway
9	PDGF signaling pathway
10	T cell activation
11	Angiogenesis
12	p53 pathway feedback loops 2
13	B cell activation
14	Insulin/IGF pathway-protein kinase B signaling cascade
15	Ras Pathway
16	Apoptosis signaling pathway
17	PI3 kinase pathway
18	PDGF signaling pathway
19	T cell activation
20	Angiogenesis
21	p53 pathway feedback loops 2

Table S2. Determination of biological processes and relevant pathways from predicted targets of miR-let-7 family members. A: Biological processes significantly (p<0.001) enriched in the predicted targets of miR-let-7 family members

S.N.	Biological process	Number of genes from our predicted target list
1	metabolic process	394.48
2	primary metabolic process	330.91
3	biological regulation	144.79
4	developmental process	127.85
5	cellular process	281.56
6	cellular protein modification process	55.1
7	protein metabolic process	134.41
8	cell communication	152.42
9	transcription, DNA-dependent	87.06
10	transcription from RNA polymerase II promoter	86.57
11	localization	123.93
12	regulation of biological process	96.96
13	regulation of nucleobase-containing metabolic process	71.19
14	protein transport	58.89
15	intracellular protein transport	58.22
16	protein phosphorylation	25.14
17	RNA metabolic process	109.35
18	regulation of transcription from RNA polymerase II promoter	66.78
19	transport	120.94
20	regulation of liquid surface tension	2.01
21	nucleobase-containing compound metabolic process	156.16
22	response to pheromone	14.62
23	anatomical structure morphogenesis	30
24	homeostatic process	6.11
25	cellular component organization	46.54
26	receptor-mediated endocytosis	9.45
27	system development	73.29
28	cellular component organization or biogenesis	50.02
29	mesoderm development	46.23
30	cellular component morphogenesis	28
31	vesicle-mediated transport	41.64
32	cell adhesion	38.61
33	biological adhesion	38.61
34	phosphate-containing compound metabolic process	23.31
35	cation transport	27.06
36	cell cycle	65.67

37	cell-cell adhesion	21.4

Table S2B: Relevant Biological pathway significantly from the predicted targets of the miR-let-7s

S.N.	Biological pathway
1	Gonadotropin releasing hormone receptor pathway
2	PDGF signaling pathway
3	Integrin signalling pathway
4	Insulin/IGF pathway-protein kinase B signaling cascade
5	Apoptosis signaling pathway
6	p53 pathway by glucose deprivation
7	B cell activation
8	p53 pathway
9	T cell activation
10	TGF-beta signaling pathway
11	Alzheimer disease-amyloid secretase pathway
12	Alpha adrenergic receptor signaling pathway
13	Adrenaline and noradrenaline biosynthesis
14	Nicotine pharmacodynamics pathway
15	Toll_pathway_drosophila
16	SCW_signaling_pathway
17	MYO_signaling_pathway
18	GBB_signaling_pathway
19	DPP_signaling_pathway

Figure S4: Gene expression level of miR-let-7f, miR-let-7g and miR-let-7i in the kidneys of control, diabetic and ARB treated diabetic mice

Real time PCR analysis using specific primers in the kidney of control, DM, ARB treated diabetic group. N=6 were analyzed in each group. Hs_RNU6 was used as internal control to normalize the expression data. Data in the graph are presented as mean±SEM. One way Anova Tukey test was performed for calculation of statistical significance.

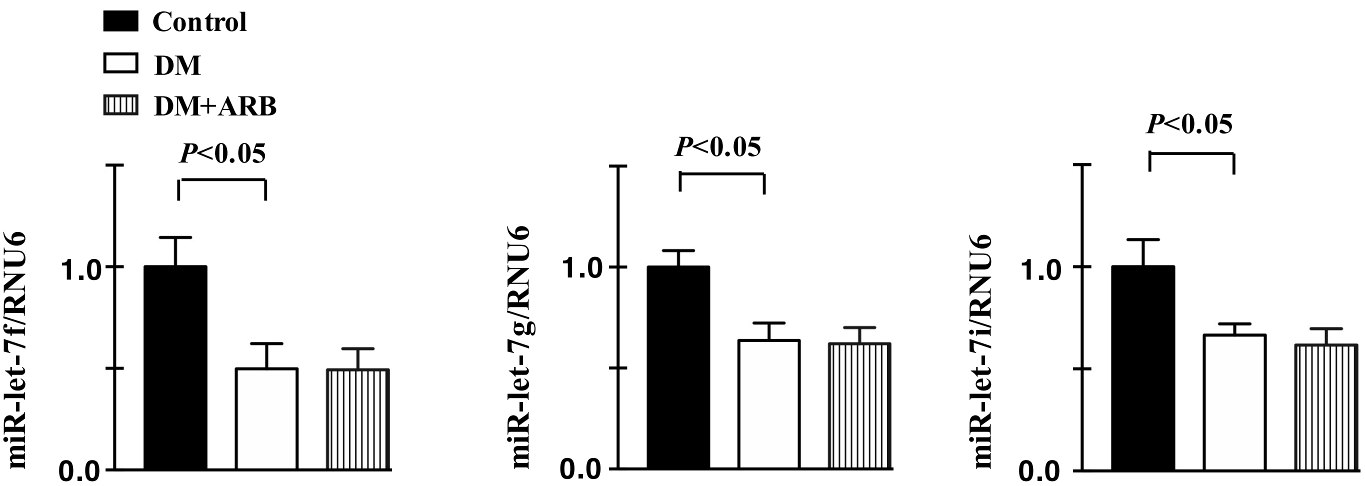

Figure S5: Gene expression level of αSMA and fibronectin in the Ang II stimulated high glucose treated HMVECs

Real time PCR analysis using specific primers in the Ang II (10 μM) stimulated cultured HMVECs. Experiment in triplicate were analyzed. 18S was used to normalize the expression data. Data in the graph are presented as mean±SEM. One way Anova Tukey test was performed for calculation of statistical significance.

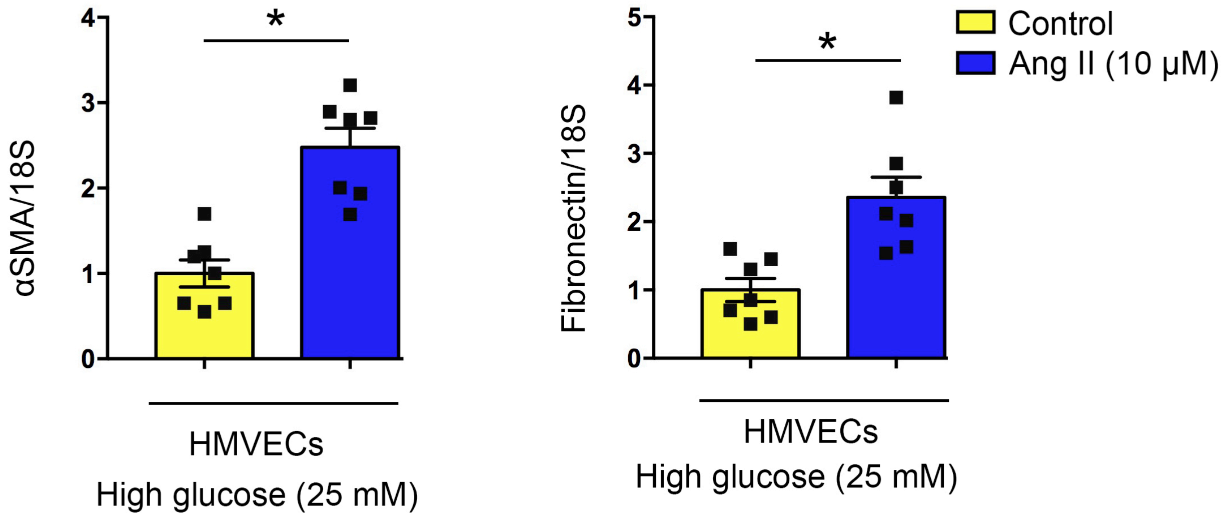

Figure S6: Gene expression analysis of DPP-4 and antifibrotic microRNAs in the POP inhibitor treated TGFβ2 stimulated HMVECs

Real time PCR analysis using specific primers in the POP inhibitor (A17092 (5 μM) **treated** TGFβ2-stimulated HMVECs. Experiment in triplicate were analyzed. 18S was used to normalize the DPP-4 whereas, HS_RNU6 was used to normalize mir-29 and miR-let-7 expression data. Data in the graph are presented as mean±SEM. N=4 were analyzed in each group. One way Anova Tukey test was performed for calculation of statistical significance.

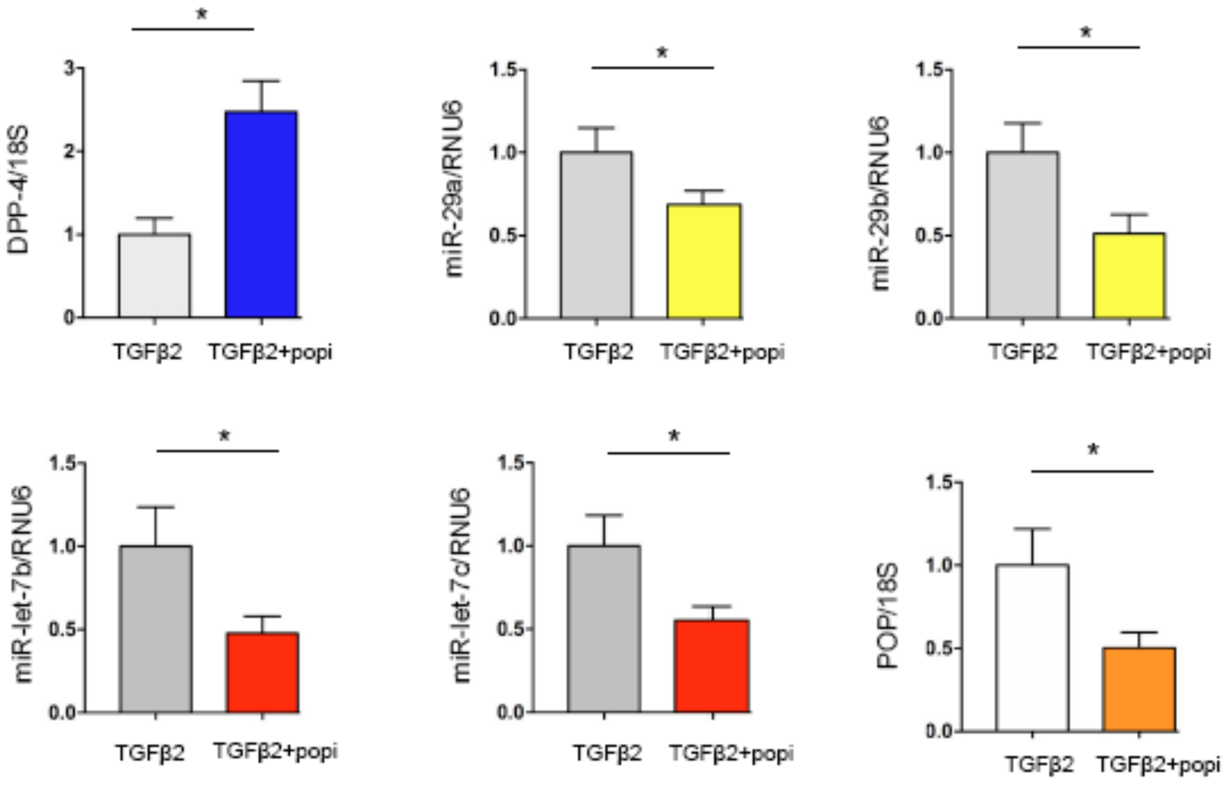

Figure S6
